# Pulmonary amyloidosis diagnosed via transbronchial lung cryobiopsy without surgical lung biopsy: A case series

**DOI:** 10.1016/j.rmcr.2022.101688

**Published:** 2022-06-20

**Authors:** Kazushi Fujimoto, Minoru Inomata, Yu Ito, Haruko Matsumoto, Ayae Saiki, Keita Sakamoto, Nobuyasu Awano, Naoyuki Kuse, Toshio Kumasaka, Takehiro Izumo

**Affiliations:** a Department of Respiratory Medicine, Japanese Red Cross Medical Center, Shibuya, Japan; b Department of Pathology, Japanese Red Cross Medical Center, Shibuya, Japan

**Keywords:** Pulmonary amyloidosis, Surgical lung biopsy, Transbronchial cryobiopsy

## Abstract

Pulmonary amyloidosis is a rare disease characterized by abnormal extracellular deposition of amyloid fibril in the lung tissue, and the identification of amyloid deposits is essential for its diagnosis. Surgical lung biopsy (SLB) is a standard diagnostic method for pulmonary amyloidosis. However, it has a relatively high post-procedural mortality rate. Recently, transbronchial lung cryobiopsy (TBLC) has been gradually used for diagnosing interstitial lung disease. However, its diagnostic efficacy for pulmonary amyloidosis has not yet been validated. Here, we describe two cases of pulmonary amyloidosis with deposition of amyloid light chain detected via TBLC. Since SLB is a high-risk procedure for the patients due to age and complications, TBLC was performed. Both patients presented with Congo red-positive amyloid deposits. One patient with localized pulmonary amyloidosis had a good clinical course without therapeutic intervention and was followed up. The other with systemic amyloidosis received chemotherapy and presented with a stable clinical course. TBLC can collect a larger pulmonary specimen for pulmonary amyloidosis than forceps biopsy and has fewer complications and a lower mortality rate than SLB. Thus, it can be a diagnostic method for pulmonary amyloidosis.

## Abbreviations

TBLC: transbronchial lung cryobiopsy
SLB: surgical lung biopsy
ILD: interstitial lung disease
AL: amyloid light chain

## 1 Introduction

Amyloidosis is a rare disease characterized by abnormal extracellular deposition of autologous misfolded proteins, known as amyloid fibril, which interferes with normal tissue and organ functions [1,2]. The respiratory system, including the lungs and bronchi, can occasionally be involved in systemic or localized amyloidosis [2]. Because the treatment for amyloidosis substantially differs between different precursors and pathogenic mechanisms, the unequivocal identification of the amyloid type is essential to prevent therapeutic errors [2].

Additionally, transbronchial lung cryobiopsy (TBLC) has been widely used for collecting lung specimens via flexible bronchoscopy, particularly for diagnosing interstitial lung disease (ILD) [3].

However, with respect to pulmonary amyloidosis diagnosis, the efficacy of TBLC compared with surgical lung biopsy (SLB) and transbronchial forceps biopsy has not been completely validated. Further, there are only a few reports about the use of TBLC for diagnosing pulmonary amyloidosis.

Here, we present two cases of pulmonary amyloid light chain (AL) amyloidosis diagnosed via TBLC without SLB.

## 2 Case presentation

### 2.1 Case 1

A 77-year-old man with a history of abnormal chest radiography finding was referred to our hospital.

The patient had hypertension, dyslipidemia, and an operative history (total resection for prostate cancer). He had a 20-pack-year smoking history but no family history of connective tissue and pulmonary diseases.

Laboratory examination showed the following: Krebs von den Lungen-6 level, 10726 U/mL; surfactant protein-D, 302 ng/mL; positivity for anti-nuclear antibody (1:320, nucleolar pattern); and negativity for anti-scl-70 antibody, anti-RNA polymerase 3 antibody, and anti-centromere antibody.

**Fig. 1:**
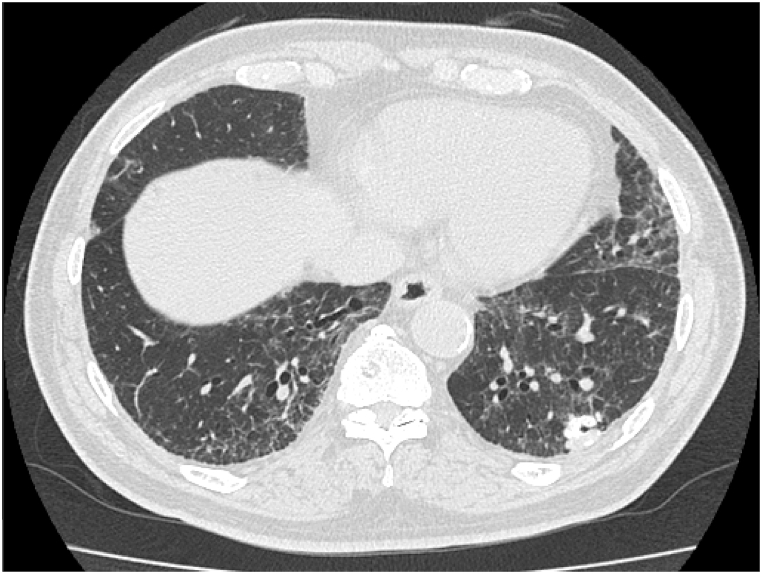
Chest computed tomography scan revealed ground-glass opacity in the basal area of both lungs and nodule in the left lower lobe.

Chest radiography revealed ground-glass opacity in the bilateral lower lung fields, and a chest computed tomography scan revealed ground-glass opacity in the basal area of both lungs and a nodule in the left lower lobe (Fig. 1). The pulmonary function test result was normal.

**Fig. 2:**
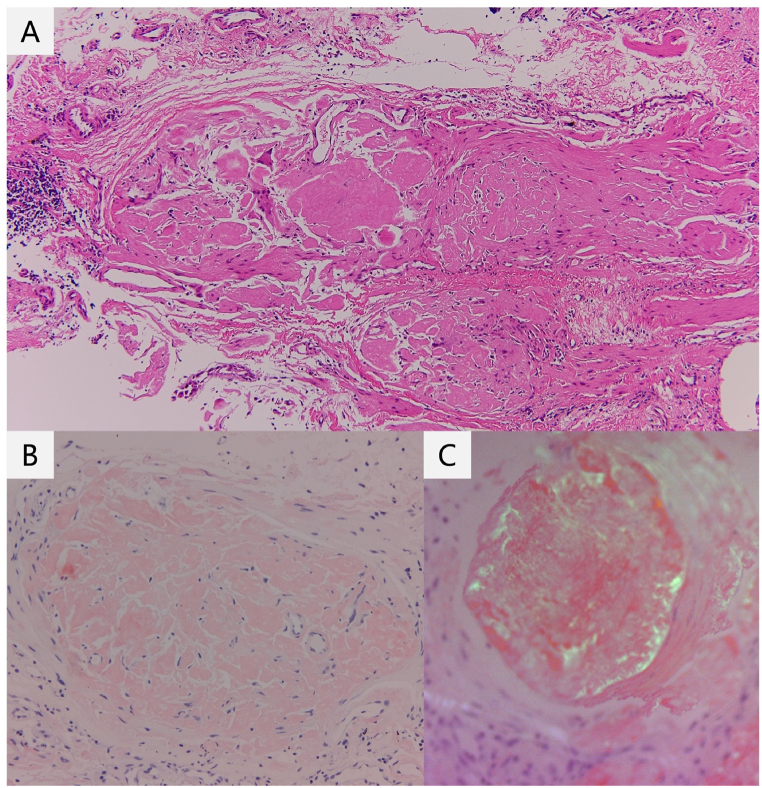
Histological findings of the lung specimen collected via transbronchial cryobiopsy (2A). Results showed the deposition of Congo red-stained AL (2B) with birefringence using polarized light (2C). (For interpretation of the references to colour in this figure legend, the reader is referred to the Web version of this article.)

Flexible bronchoscopy revealed normal intraluminal findings. Following this, a 51% recovery rate was achieved using bronchoalveolar lavage from the superior lingular segment; it indicated 59% lymphocytes and no malignant cells. The finding of hyperlymphocytosis in bronchoalveolar lavage fluid was considered primarily due to the etiology of ILD. Culture samples collected using bronchoscopy tested negative for common bacteria, mycobacteria, and fungus. Prior to TBLC, we introduced a 1.4-mm 20-MHz radial probe (PB2020-M; Fujifilm) with a guide sheath into the target bronchus and checked whether any blood vessel ran parallel to the bronchus. We obtained the endobronchial ultrasonographic view of “adjacent to the lesion” from B^9^a. We performed TBLC with a 1.9 × 1150-mm flexible cryoprobe (ERBECRYO 2 system; Erbe Elektromedizin GmbH, Tubingen, Germany) introduced through the guide sheath using an endobronchial balloon as an endobronchial blocker. Freezing time was set as 6 s. There was minor bleeding, which was easily controlled with an endobronchial blocker and an intrabronchial injection of an epinephrine-saline mixture (1-mL epinephrine with 19-mL saline). On high-resolution computed tomography (HRCT), the nodule was located in the left lateral basal segment (S^9^), and the TBLC sites were left B^8^b, B^9^a, and B^9^b. The pathological findings of the TBLC-collected specimen from left B^9^a showed the deposition of Congo red-stained AL with birefringence under polarized light (Fig. 2A–C). Except for the lesion of pulmonary amyloidosis, ground-glass opacities in both lungs were diagnosed as interstitial pneumonia, most likely due to chronic hypersensitivity pneumonia or interstitial pneumonia with autoimmune features. An additional biopsy was performed using bone marrow and subcutaneous adipose tissue samples, and the result was negative. Hence, the patient was diagnosed with localized nodular pulmonary amyloidosis. Since the condition was asymptomatic, he refused medical intervention. During follow-ups, he was found to have a good clinical condition.

### 2.2 Case 2

A 62-year-old man with systemic AL amyloidosis was admitted to our hospital because of fever. The patient had noticed bilateral leg edema one year ago, and an annual medical check-up revealed an abundance of protein in the urine. He was referred to a different hospital for further investigation and a renal biopsy, followed by the diagnosis of AL amyloidosis. Amyloidosis was found in the heart, intestines, and tongue, among other organs. The Department of Hematology in our hospital was referred to the patient. In the hematology department, he received autologous peripheral blood stem cell transplantation after receiving combined melphalan and dexamethasone therapy. Following this, he was treated with six courses of combined melphalan and dexamethasone therapy. Laboratory tests showed elevated C-reactive protein levels at 5.29 mg/dL and normal cell count. He presented with mild renal impairment (serum creatinine level of 1.08 mg/dL). Moreover, his immunoglobulin G level decreased to 753 mg/dL. Additionally, we tested him for beta-D-glucan, *Aspergillus* galactomannan antigen, cytomegalovirus antigen, and anti-glycopeptidolipid-core IgA for *Mycobacterium avium* and performed interferon-gamma release assay; however, these tests were negative.

**Fig. 3:**
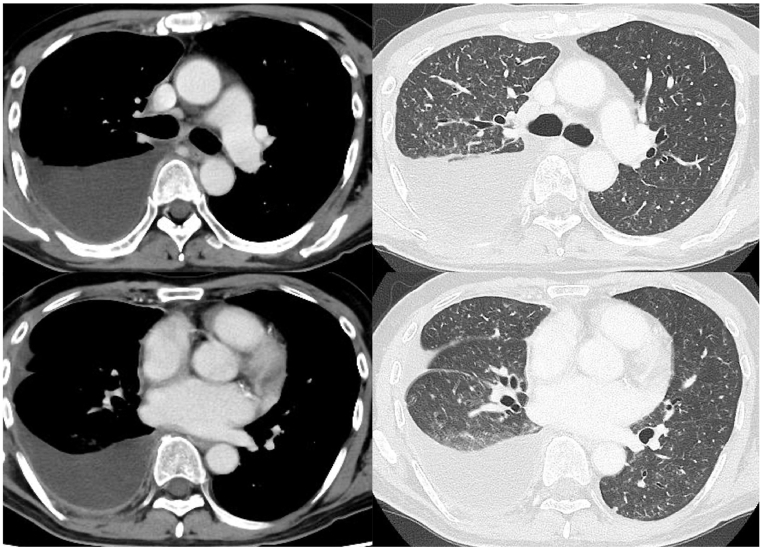
Chest computed tomography scan revealed right pleural effusion and multiple micronodules with a random distribution in both lung fields.

Chest radiography showed pleural effusion in the right thoracic cavity and bilateral ground-glass opacity. Chest computed tomography scan revealed right pleural effusion and multiple micronodules with a random distribution in both lung fields (Fig. 3).

Transthoracic echocardiography revealed a low ejection fraction with moderate left ventricular hypertrophy and granular myocardial appearance.

Hence, miliary tuberculosis was suspected. Next, sputum, urine, and blood smear assessments were performed to detect mycobacterium. However, Ziehl–Neelsen staining showed no acid-fast bacillus.

Exudates were discovered during a thoracocentesis, although the culture of pleural effusion samples did not show common bacteria or acid-fast bacteria.

Although there was a risk of TBLC in patients with lung volume loss and a low ejection fraction, we considered that the risk of cancer was significantly higher than that of TBLC. The patient then underwent flexible bronchoscopy to examine the cause of multiple pulmonary micronodules. The bronchus surface was normal on flexible bronchoscopy. We collected lung specimens from the right B^2^b, B^3^a, and B^4^a via both forceps biopsy and cryobiopsy. We used a guide sheath under radiographic guidance and performed TBLB and TBLC through the same guide sheath left behind in the target bronchus. This approach allowed us to take both TBLB and TBLC specimens theoretically from the same place in the lung, minimizing the difference in the sampling error between TBLB and TBLC. The forceps biopsy specimen showed no microbiological findings based on gram staining, Ziehl–Neelsen staining, and Grocott staining. Further, the specimen cultures had negative results.

**Fig. 4:**
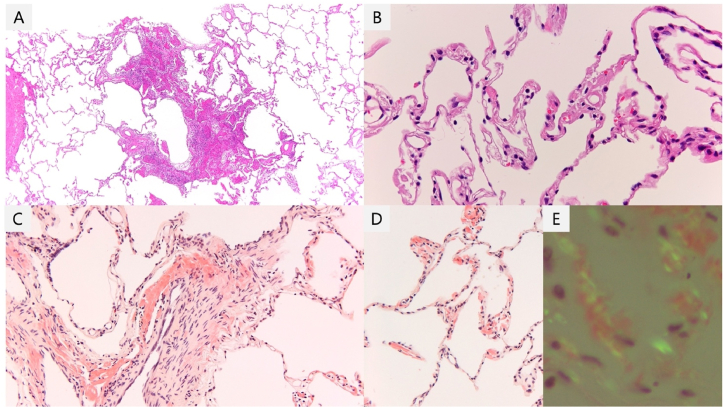
Histological analysis of the lung specimen collected via TBLC showed the deposition of fibrin and infiltration of lymphocytes, eosinophils, and macrophages in the alveolar space (4A). Moreover, it revealed mild thickening of the alveolar wall (4B) and pulmonary arterioles (4C), both of which had amyloid deposition with positive Congo-red staining findings (4C, 4D) accompanied by birefringence using polarized light (4E). (For interpretation of the references to colour in this figure legend, the reader is referred to the Web version of this article.)

The pathological findings of the specimen collected via forceps biopsy showed no evidence of amyloidosis. However, those taken via TBLC revealed mild thickening of the alveolar wall and pulmonary arterioles, both of which had amyloid deposition with a positive finding on Congo-red staining (Fig. 4A–D) accompanied by birefringence using polarized light (Fig. 4E).

The lung lesion was associated with systemic amyloidosis. The patient then received continuous chemotherapy for systemic amyloidosis with melphalan and dexamethasone, and his disease behavior has been stabilized for one year to date.

## 3 Discussion

Amyloidosis is commonly classified as systemic or local, and systemic amyloidosis is often fatal if not effectively treated [2]. There are several types of amyloid fibrils. The most common types are AL protein and amyloid-associated type of amyloid fibril proteins, which are typically associated with multiple myeloma and chronic inflammatory conditions, respectively [4]. The radiological characteristics of amyloidosis vary and are typically nonspecific. Hence, the diagnosis of pulmonary amyloidosis based on radiological features alone is challenging [5]. An amyloid fibril must exhibit affinity for Congo red with green, yellow, or orange birefringence visualized using a polarized light [1]. The clinical management completely depends on the type of protein deposited. Hence, a high-risk therapeutic intervention is commonly required [6].

The respiratory system, including the lungs and bronchi, can be involved occasionally in systemic and local amyloidosis [2]. Lung involvement is relatively common but is rarely symptomatic [2,7]. Lung involvement may manifest as diffuse reticulonodular interstitial thickening, consolidation, or solitary or multiple parenchymal nodules that may calcify, cavitate, and slowly enlarge. Pulmonary amyloidosis is often classified as tracheobronchial amyloidosis, diffuse alveolar-septal amyloidosis, and nodular pulmonary amyloidosis [8].

The current cases had two notable findings. First, to the best of our knowledge, this is the first case series about pulmonary amyloidosis with pathological images of TBLC specimens. Second, TBLC might be more acceptable for the diagnosis of pulmonary amyloidosis similar to other ILDs other than SLB or forceps biopsy.

Although TBLC has been widely used for lung biopsy particularly in ILD diagnosis [3], no case had pathological images of pulmonary amyloidosis diagnosed via TBLC. This procedure collects lung specimens via cryoadhesion between a frozen-tipped probe and adjacent lung parenchyma under bronchoscopic guidance. As the evidence for this procedure continues to grow, some centers are no longer using conventional SLB and prefer the less invasive type of cryobiopsy. As only approximately 10 years have passed since cryobiopsy first emerged [3], its use has not been completely adopted worldwide. However, some guidelines and statements suggested TBLC for newly detected ILDs in the past. Our recommendations for patients with newly detected ILDs without a HRCT pattern of usual interstitial pneumonia (UIP) are neither for nor against TBLC, according to the ATS/ERS/JRS/ALAT guidelines for idiopathic pulmonary fibrosis [9]. Cases 1 and 2 have HRCT patterns of “indeterminate for UIP” and “an alternate diagnosis,” respectively, which we considered to be far from the “UIP pattern” in HRCT. Hence, we performed TBLC in these two cases. We recommend obtaining biopsy samples from at least two sites with the tip of the cryoprobe located 1 cm from the pleura and using a fluoroscope, endobronchial blockers, and a 1.9-mm-diameter cryoprobe, according to the CHEST guidelines for TBLC in patients with ILD [10]. We performed TBLC in these cases, as well as other TBLC cases in our hospital, according to the recommendations of the CHEST guidelines. Ussavarungsi et al. retrospectively analyzed 74 patients with ILD diagnosed using TBLC [11]. In their study, one patient was diagnosed with pulmonary amyloidosis; this is the only reported case of pulmonary amyloidosis identified via TBLC. TBLC should be used in more cases to validate its diagnostic accuracy by accumulating evidence.

Despite the advantages of cryobiopsy, there are still concerns about its safety and diagnostic accuracy [12]. In some cases, a small number of specimens can be obtained by a conventional forceps biopsy to diagnose pulmonary amyloidosis. However, TBLC can obtain a larger sample than a forceps biopsy, and the heterogeneity of amyloid deposition in lung specimens can be addressed by TBLC. To the best of our knowledge, there has been no report on the comparison between the diagnostic yields of TBLB and TBLC in pulmonary amyloidosis. In diagnosing ILD, it has been reported that the diagnostic yield of TBLC is better than that of TBLB because of the larger sample size and fewer sampling artifacts [13]. The conventional forceps biopsy, which obtains a smaller specimen, is highly affected by crush artifacts, and the ratio of artifact-free area to the overall specimen area is less than that in the TBLC specimen. Therefore, it can be used to diagnose pulmonary amyloidosis. Furthermore, the adverse effects of TBLC are nearly identical to those of forceps biopsy [13].

The overall in-hospital mortality rates among patients with ILD are 6.4% for SLB, 1.7% for scheduled procedures, and up to 16% for emergent therapies, according to Hutchinson JP et al. [14]. Male sex, older age, the presence of comorbidities, open surgery, and idiopathic pulmonary fibrosis or connective tissue disease correlated with ILD were all risk factors for high mortality rate. Furthermore, using the updated Charlson Comorbidity Index, this study revealed the risk of in-hospital mortality after a scheduled SLB for ILD, which was classified according to age and comorbidity [14]. The risk of 30-day in-hospital mortality was 10.1% (95% confidence interval: 7.33%–13.83%) in case 1 and 5.4% (95% confidence interval: 4.02–7.12) in case 2. Moreover, the most common SLB complications were postoperative pneumothorax (8.7%), pulmonary collapse (6.4%), pneumonia (5.8%), pleural effusion (3.2%), and respiratory failure (3.1%). In contrast, the procedural mortality rate of TBLC was 0.3%, which was lower than that of SLB [15]. Major procedural complications were bleeding and pneumothorax, which occurred in 22% and 1.4% of patients, respectively. The bleeding was mild-to-moderate and could be treated with bronchial balloon blockers [11]. TBLC was used to diagnose these representative cases. Hence, SLB was not required because of its high mortality and complication rates. TBLC can be a safer approach for diagnosing pulmonary amyloidosis than SLB.

## 4 Conclusion

We present two cases of pulmonary amyloidosis with deposition of AL detected via TBLC without SLB. The type of amyloidosis should be confirmed to facilitate appropriate treatment. TBLC can collect a larger pulmonary specimen for pulmonary amyloidosis compared with forceps biopsy, and it has fewer complications and mortality rate than SLB. Hence, it can be a diagnostic method for pulmonary amyloidosis.

## Learning points

• We described two cases of pulmonary amyloidosis with deposition of AL detected using TBLC without SLB.
• We presented the pathological images of pulmonary amyloidosis whose specimens were taken via TBLC.
• TBLC can collect a larger pulmonary specimen for pulmonary amyloidosis than forceps biopsy and has fewer complications and a lower mortality rate than SLB.

## Funding

This research did not receive any specific grant from funding agencies in the public, commercial, or not-for-profit sectors.

## Declaration of competing interest

None.
